# A. V. M. A.

**Published:** 1903-05

**Authors:** 


### A. V. M. A.

SECRETARY REPP, of the A. V. M. A., under date of April 8th, makes the following announcement relative to the railroad rates for the Ottawa meeting:

The Canadian Pacific Railway, the Grand Trunk Railway, and the Canada Atlantic Railway have granted a special rate of one and one-third fare for the round trip, certificate plan, for 50 or more in attendance, and one and two-thirds fare for 49 or less in attendance. The New England Passenger Association and the Trunk Line Passenger Association have granted a rate of one and one-third fare, certificate plan, for 100 or more in attendanee, and the Southeastern Passenger Bureau has refused to grant a special rate from its territory. Those attending from this latter territory should purchase a ticket to the nearest point at which certificates are kept in the territory of the Trunk Line Association or the Central Passenger Association, if this latter should grant a special rate, as it is expected to do, and then purchase a ticket from this point to Ottawa and take certificate therefor. It is confidently expected that special rates will later be granted by the Central and Western Passenger Association, and perhaps by the Southwestern, from part of its territory, at least. Report will be made through the journals as soon as these Associations take action. Full information will accompany the programme. This announcement is made so that our members and others may know at the earliest possible moment what to look for in the way of rates, as this will doubtless have an important bearing in deciding the question whether they will attend the meeting or not.

